# Single nucleotide polymorphisms (SNPs) of *ERCC2*, *hOGG1*, and *XRCC1* DNA repair genes and the risk of triple-negative breast cancer in Polish women

**DOI:** 10.1007/s13277-013-1461-0

**Published:** 2014-01-09

**Authors:** Beata Smolarz, Marianna Makowska, Dariusz Samulak, Magdalena M. Michalska, Ewa Mojs, Maciej Wilczak, Hanna Romanowicz

**Affiliations:** 1 Laboratory of Molecular Genetics, Department of Pathology, Institute of Polish Mother’s Memorial Hospital, Rzgowska 281/289, 93-338 Lodz, Poland; 2 Regional Hospital in Lodz, Łódź, Poland; 3 Department of Obstetrics and Gynaecology, Regional Hospital in Kalisz, Kalisz, Poland; 4 Cathedral of Mother’s and Child’s Health, Poznan University of Medical Sciences, Poznań, Poland; 5 Clinic of Gynaecological Surgery, Poznan University of Medical Sciences, Poznań, Poland; 6 Department of Clinical Psychology, Poznan University of Medical Sciences, Poznań, Poland; 7 Department of Medical Education, Poznan University of Medical Sciences, Poznań, Poland; 8 University of Computer Sciences and Skills, Lodz, Poland

**Keywords:** Triple-negative breast cancer, *ERCC2*, *hOGG1*, *XRCC1*, Genes, Genetic polymorphism

## Abstract

Triple-negative breast cancer (TNBC) refers to about 15–20 % of all breast cancer cases. It is characterized by worse clinical outcome, poor prognosis, and absence of prognostic indicators. Several polymorphisms in the nucleotide excision repair (NER) and base excision repair (BER) gene have been extensively studied in association with various human cancers. The aim of this study was to evaluate the role of the *hOGG1*-Ser326Cys (rs13181), *XRCC1*-Arg194Trp (rs1799782), and *ERCC2*-Lys751Gln (rs13181) gene polymorphisms with clinical parameters and the risk for development of triple-negative breast cancer. Our research included 70 patients with TNBC and 70 healthy controls. Gene polymorphisms were genotyped by the PCR-RFLP (restriction fragment length polymorphism) method. The genotype distributions were contrasted by the chi-square test, and the significance of the polymorphism was assessed by multiple logistic regression producing odds ratios (ORs) and 95 % confidence intervals (CIs). In the present work, a relationship was identified between *ERCC2*-Lys751Gln polymorphism and the incidence of triple-negative breast cancer. An association was observed between triple-negative breast carcinoma occurrence and the presence of Gln/Gln genotype (OR = 5.71 (2.12–5.43), *p* = 0.0007). A tendency for an increased risk of TNBC was detected with the occurrence of 751Gln allele of *ERCC2 p*olymorphism. No significant associations between Ser326Cys and Arg194Trp genotype and TNBC were observed. We suggest that the Lys751Gln polymorphism of the *ERCC2* gene may be risk factors for triple-negative breast cancer development in Polish women.

## Introduction

Carcinoma of the breast is the most common cause of cancer deaths among women worldwide. Despite a decline in incidence since 2003, in 2008, nearly 1,400,000 new cases of breast cancer were diagnosed, and there were about 450,000 women who died from this disease [1, 2].

Currently, more women survive due to earlier diagnosis and better therapy. Breast cancer classification is in constant evolution, as advances in molecular pathology as well as immunohistochemical staining allow researchers to define the molecular heterogeneity of different disease subtypes and to guide the selection of appropriate treatment.

The triple-negative phenotype, defined as the lack of estrogen receptor (ER), progesterone receptor (PR), and human epidermal growth factor receptor-2 (HER-2) expression, represents approximately 15–20 % of breast cancer cases and has a worse clinical outcome and prognosis than other breast cancer subtypes [2–6]. As a group, triple-negative breast cancer (TNBC) is frequently associated with development of distant metastasis, shorter survival, and a higher mortality rate than other disease subtypes. Most recurrences are observed during the first and third years after therapy, and most deaths take place in the first 5 years, even after a strict therapeutic regimen. The triple negativity represents an independent factor for poor prognosis evaluation for breast cancer [2–6].

Breast cancer may be associated with the high exposure of breast tissue to exo- and endogenous estrogens. Estrogens produce DNA bulky adducts and oxidative base damages which are removed in nucleotide excision repair (NER) and base excision repair (BER) systems. The reaction of breast cells to DNA damage may be very important for their susceptibility to cancer development. This reaction is executed mainly by DNA repair, which can be modulated by the variability in the genes encoding DNA repair proteins.

A NER system removes short DNA oligonucleotides containing a damaged base [7]. NER recognizes bulky lesions caused by carcinogenic compounds and covalent linkages between adjacent pyrimidines resulting from UV exposure. NER is further classified into global genome repair (GG-NER) that occurs everywhere in the genome and transcription-coupled repair (TCR), which removes lesions in the transcribed strand of active genes. NER is a multistep process involving multiple proteins such as ERCC1, *ERCC2*, ERCC3, ERCC4, PCNA, RPA, XPA, and p53.

BER is critically important for repairing base damage induced by reactive oxygen species (ROS). BER corrects small DNA alterations that do not distort the overall structure of DNA helix, such as oxidized bases, or incorporation of uracil. BER is initiated by DNA glycosylases, which cleave N-glycosylic bond of damaged bases leaving apurinic/apyrimidinic site (AP site) [8, 9].

A damaged base is recognized by a specific glycosylase, which cleaves the bond between the base and sugar, creating an abasic site, which is cleaved by an endonuclease. Resulting gap is filled by polβ, and the remaining nick is sealed by DNA ligase LIG1 or LIG3 complexed with XRCC1.

Because NER and BER are involved in removing a substantial number of DNA damages, which can contribute to the genome instability, it is reasonable to check whether variability in the genes coding for BER and NER products may be associated with TNBC.

In the present work, we analyzed an association between TNBC and three SNPs occurring in two BER and NER genes: *hOGG1*-Ser326Cys (rs13181), *XRCC1-*Arg194Trp (rs1799782) and *ERCC2*-Lys751Gln (rs13181), respectively. These polymorphisms have been correlated with various cancers [10–24], but little is known about their association with TNBC.

## Materials and methods

### Patients

In the present study, paraffin-embedded tumor tissues were obtained from 70 women with triple-negative breast carcinoma, treated at the Department of Oncology, Institute of Polish Mother's Memorial Hospital, Lodz, Poland, between 2000 and 2013. Clinical data for the patients and histological data were registered. The age of the patients ranged from 36 to 68 years (mean age 46.2 ± 10.12). The median follow-up of patients at the time of analysis was 38 months (range 2–70 months). The average tumor size was 20 mm (range 17–32 mm). All the tumors were graded by a method, based on the criteria of Scarf–Bloom–Richardson. There were 20 tumors of stage I, 45 of stage II, and 5 of stage III in total. The demographic data and the pathologic features of the patients are summarized in Table 1. Samples from age-matched, cancer-free women (*n* = 70) served as the control (mean age 45.41 ± 18.21). Control samples that consisted of DNA were extracted from normal breast tissue. Normal breast specimens were obtained from patients who had undergone biopsy for benign lesions. The study was approved by the Local Ethics Committee of the Institute of Polish Mother's Memorial Hospital, Lodz, Poland, and each patient gave a written consent.

**Table 1:** Characteristics of the study population (*n* = 70) triple-negative breast cancer patients

	Triple-negative breast cancer patients, *n* (%)
Scarf–Bloom–Richardson stage	
I	20 (29)
II	45 (64)
III	5 (7)
Tumor size grade	
T1	8 (11)
T2	40 (57)
T3	18 (26)
T4	4 (6)
Lymph node status	
N0	32 (46)
N1	12 (17)
N2	14 (20)
N3	7 (10)
N4	5 (7)

### DNA isolation

The cancerous and noncancerous breast tissue samples were fixed routinely in formaldehyde, embedded in paraffin, cut into thin slices, and stained with hematoxylin/eosin for pathological examination. DNA for analysis was obtained from an archival pathological paraffin-embedded tumor and noncancerous breast samples which were deparaffinized in xylene and rehydratated in ethanol and distilled water. In order to ensure that the chosen histological material is representative for cancerous and noncancerous tissue, every tissue sample qualified for DNA extraction was initially checked by a pathologist. DNA was extracted from the material using commercially available QIAamp Kit (Qiagen GmbH, Hilden, Germany) DNA purification kit according to the manufacturer's instruction.

### Determination of ERCC2 genotype

Polymorphism Lys751Gln of the *ERCC2* gene was determined by PCR-RFLP, using primers (forward 5′-CTGCTCAGCCTGGAGCAGC-3′ and reverse 5′-ACTGTCACTAGTCTCACCAG-3′). The PCR was carried out in a PTC-100 TM (MJ Research, INC) thermal cycler. PCR amplification was performed in the final volume of 25 μl of reaction mixture, which contained 100 ng of genomic DNA, 0.2 μmol of each primer (ARK Scientific GmbH Biosystems, Darmstad, Germany), 2.5 mM of MgCl_2_, 1 mM of dNTPs, and 1 U of Taq polymerase (Qiagen GmbH, Hilden, Germany). PCR cycle conditions were the following: 95 °C for 30 s, 62 °C for 30 s, and 72 °C for 30 s, repeated in 35 cycles. PCR products were electrophoresed in a 2 % agarose gel and visualized by ethidium bromide staining. The cleavage with *Pst*I (Fermentas, Vilnius, Lithuania) produced fragments of 161, 161/120/41, and 120/41 bp corresponding to the Lys/Lys, Lys/Gln, and Gln/Gln genotypes of the *ERCC2* gene, respectively.

### Determination of hOGG1 genotype

Polymorphism Ser326Cys of the *hOGG1* gene was determined by PCR-RFLP, using primers (5′-GGAAGGTGCTTGGGGAAT-3′ and 5′-ACTGTCACTAGTCTCACCAG-3′). The 25-μL PCR mixture contained about 100 ng of DNA, 12.5 pmol of each primer, 0.2 mmol/L of dNTPs, 2 mmol/L of MgCl_2_, and 1 U of Taq DNA polymerase. PCR products were electrophoresed in a 2 % agarose gel and visualized by ethidium bromide staining. Only one 100-bp fragment was seen in subjects with the Cys/Cys genotype. In subjects with the Ser/Cys genotype, two bands of 100 and 200 bp were seen, whereas in those subjects homozygous for the Ser variant (Ser/Ser), only one 200-bp PCR fragment is seen. All PCR was carried out in a DNA thermal cycler (GeneAmp PCR System 2400; Perkin-Elmer, Norwalk, CT, USA). After an initial denaturation at 95 °C for 5 min, 35 cycles of amplification with denaturation at 95 °C for 30 s, annealing at 56 °C for 30 s, and extension at 72 °C for 30 s were performed, followed by a final extension step of 7 min at 72 °C. The PCR product was digested overnight with 1 U of *Sat*I (Fermentas, Vilnius, Lithuania) at 37 °C.

### Determination of XRCC1 genotype

Polymorphism Arg194Trp of the *XRCC1* gene was determined by PCR-RFLP, using primers (forward 5′-GCCCGTCCCAGGTA-3′, reverse 5′-AGCCCCAAGACCCTTTCACT-3′).

The PCR was carried out in a PTC-100 TM (MJ Research, Inc.) thermal cycler. PCR amplification was performed in the final volume of 25 μl of reaction mixture, which contained 100 ng of genomic DNA, 0.2 μmol of each primer (ARK Scientific GmbH Biosystems, Darmstad, Germany), 2.5 mM of MgCl2, 1 mM of dNTPs, and 1 U of Taq Polymerase (Qiagen GmbH, Hilden, Germany). PCR cycle conditions were the following: 95 °C for 30 s, 62 °C for 30 s, and 72 °C for 40 s, repeated in 35 cycles. After digestion with *Pvu*II (New England Biolabs, Ipswich, MA, USA) for 4 h at 37 °C, the samples were run on 2 % agarose gel and visualized by ethidium bromide staining. The cleavage of the *XRCC1* fragment with *Pvu*II (New England Biolabs, Ipswich, MA, USA) produced bands of 292/174/21, 313/292/174/21, and 313/174 bp corresponding to the Arg/Arg, Arg/Trp, and Trp/Trp genotypes, respectively.

### Statistical analysis

The allelic frequencies were estimated by gene counting, and the genotypes were scored. The observed numbers of each *hOGG1*, *XRCC1*, and *ERCC2* genotype were compared with those expected for a population in Hardy–Weinberg equilibrium by using the chi-square test. Genotype frequencies in the study cases and the controls were compared by the chi-square test. Genotype-specific risks were estimated as odds ratios (ORs) with associated 95 % confidence intervals (CIs) by unconditional logistic regression. *p* values < 0.05 were considered significant. All the statistical analyses were performed, using the STATISTICA 6.0 software (Statsoft, Tulsa, OK, USA).

## Results

All the recruited TNBC samples (*n* = 70) and control (*n* = 70) were successfully genotyped for the *ERCC2*, *hOGG1*, and *XRCC1* polymorphisms. From the PCR analysis, all patients were classified into three genotypes of the *ERCC2* polymorphism: Lys/Lys, Lys/Gln and Gln/Gln, and *hOGG1* polymorphism; Ser/Ser, Ser/Cys and Cys/Cys, and *XRCC1* polymorphism; and Arg/Arg, Arg/Trp, and Trp/Trp genotypes.

It can be seen from Table 2 that there are significant differences in the frequency of *ERCC2-*Lys751Gln genotypes (*p* < 0.05) between the two investigated groups. A weak association was observed between triple-negative breast carcinoma occurrence and the presence of Gln/Gln genotypes. Variant 751Gln allele of *ERCC2* increased cancer risk. In case of the Lys751Gln polymorphism of *ERCC2* gene, the distribution of the genotypes in the patients differed significantly from the one expected from the Hardy–Weinberg equilibrium (*p* < 0.05).

**Table 2:** Distribution of Lys/Lys, Lys/Gln, and Gln/Gln genotypes and frequencies of the Lys and Gln alleles of the *ERCC2* gene in patients with triple-negative breast cancer and controls

*ERCC2*-Lys751Gln	TNBC patients (*n* = 70)		Controls (*n* = 70)		OR (95 % CI)^a^	*p* ^b^
	Number	(%)	Number	(%)		
Lys/Lys	10	14	16	23	1.00 Ref	
Lys/Gln	10	14	40	57	0.40 (0.14–1.14)	0.144
Gln/Gln	50	72	14	20	**5.71** (**2.12**–**5.34**)	**0.0007**
Lys	30	21	72	51	1.00 Ref	
Gln	110	79	68	49	**3.88** (**2.30**–**6.55**)	<**0.0001**

Data in boldface are statistically significant

^a^Crude odds ratio (confidence interval at 95 %)

^b^Chi-square

No statistically significant differences were observed in genotype frequencies of *hOGG1-*Ser326Cys and *XRCC1*-Arg194Trp polymorphisms between the control group and the TNBC patients (see Tables 3 and 4). Among the patients, all genotype distributions did not differ significantly (*p* > 0.05) from those expected from the Hardy–Weinberg equilibrium.

**Table 3:** Distribution of Ser/Ser, Ser/Cys, and Cys/Cys genotypes and frequencies of the Arg and His alleles of the *hOGG1* gene in patients with triple-negative breast cancer and controls

hOGG1-Ser326Cys	TNBC patients (*n* = 70)		Controls (*n* = 70)		OR (95 % CI)^a^	*p* ^b^
	Number	(%)	Number	(%)		
Ser/Ser	16	23	16	23	1.00 Ref	
Ser/Cys	39	56	38	54	1.02 (0.45–2.34)	0.888
Cys/Cys	15	21	16	23	0.93 (0.34–2.51)	0.887
Ser	71	51	70	50	1.00 Ref	
Cys	69	49	70	50	0.97 (0.60–1.55)	1.000

^a^Crude odds ratio (confidence interval at 95 %)

^b^Chi-square

**Table 4:** Distribution of Arg/Arg, Arg/Trp, and Trp/Trp genotypes and frequencies of the Thr and Met alleles of the *XRCC1* gene in patients with triple-negative breast cancer and controls

XRCC1-Arg194Trp	TNBC patients (*n* = 70)		Controls (*n* = 70)		OR (95 % CI)^a^	*p* ^b^
	Number	(%)	Number	(%)		
Arg/Arg	20	29	15	21	1.00 Ref	
Arg/Trp	31	44	39	56	0.59 (0.26–1.35)	0.301
Trp/Trp	19	27	16	23	0.89 (0.34–2.28)	1.000
Arg	71	51	69	49	1.00 Ref	
Trp	69	49	71	51	0.95 (0.59–1.50)	0.920

^a^Crude odds ratio (confidence interval at 95 %)

^b^Chi-square

Histological grading was related to *ERCC2*-Lys751Gln, *hOGG1*-Ser326Cys, and the *XRCC1*-Arg194Trp polymorphisms. Histological stages were evaluated in all the cases (*n* = 70). There were 20 cases in stage I, 45 cases in stage II, and 5 cases in stage III. Stages II and III were accounted together for statistical analysis (see Table 5). No differences were observed in those groups regarding either *ERCC2-*Lys751Gln genotype or allele distributions. Some correlation was observed between the *hOGG1-*Ser326Cys and *XRCC1*-Arg194Trp polymorphisms and TNBC invasiveness. An increase was observed regarding Ser/Cys heterozygotes frequency (OR 2.42; 95 % CI 0.58–9.99, *p* = 0.177) and Arg/Trp heterozygotes (OR 1.89; 95 % CI 0.54–6.57, *p* = 0.479) in stage I patients, according to the Scarf–Bloom–Richardson classification. That increase was, however, not statistically significant (*p* > 0.05).

**Table 5:** Dependence of genotypes and frequencies of *ERCC2*, *hOGG1*, and *XRCC1* gene polymorphism alleles on tumor stage in triple-negative breast cancer patients (*n* = 70)

	Triple-negative breast cancer patients		OR (95 % CI)^b^	*p* ^c^
Stage^a^	I (*n* = 20)	II + III (*n* = 50)		
	Number (%)	Number (%)		
*ERCC2*-Lys751Gln				
Lys/Lys	5 (25)	5 (10)	1.00 Ref	
Lys/Gln	2 (10)	8 (16)	0.25 (0.03–1.81)	0.175
Gln/Gln	13 (65)	37 (74)	0.35 (0.08–1.41)	0.129
Lys	12 (30)	18 (18)	1.00 Ref	
Gln	28 (70)	82 (82)	0.51 (0.21–1.19)	0.182
*hOGG1*-Ser326Cys				
Ser/Ser	3 (15)	13 (26)	1.00 Ref	
Ser/Cys	14 (70)	25 (50)	2.42 (0.58–9.99)	0.177
Cys/Cys	3 (15)	12 (24)	1.09 (0.29–4.08)	0.588
Ser	20 (50)	51 (51)	1.00 Ref	
Cys	20 (50)	49 (49)	1.08 (0.18–6.43)	0.640
*XRCC1*-Arg194Trp				
Arg/Arg	5 (25)	15 (30)	1.00 Ref	
Arg/Trp	12 (60)	19 (38)	1.89 (0.54–6.57)	0.479
Trp/Trp	3 (15)	16 (32)	0.56 (0.11–2.77)	0.377
Arg	22 (55)	49 (49)	1.00 Ref	
Trp	18 (45)	51 (51)	0.78 (0.37–1.64)	0.646

^a^According to the Scarf–Bloom–Richardson criteria

^b^Crude odds ratio (confidence interval at 95 %)

^c^Chi-square

Table 6 shows the distribution of genotypes and the frequency of alleles in patients with different tumor size. A tendency for an increased risk of TNBC was observed with the occurrence of 751Gln allele of *ERCC2* polymorphism. That increase was statistically significant (*p* < 0.05). There were no differences either in the distribution of genotypes or the frequency of alleles in the group of patients with (N+) and without (N−) lymph node metastases (Table 6).

**Table 6:** *RAD51*, *XRCC2*, and *XRCC3* gene polymorphism and triple-negative breast cancer progression^a^

	TNBC patients (*n* = 70)		OR (95 % CI)^a^	TNBC patients (*n* = 70)		OR (95 % CI)^b^
	Tumor size			Node status		
	T3 + T4 (*N* = 22)	T1 + T2 (*N* = 48)		N+ (*n* = 38)	N− (*n* = 32)	
	Number (%)	Number (%)		Number (%)	Number (%)	
*ERCC2*-Lys751Gln						
Lys/Lys	1 (5)	9 (19)	1.00 Ref	5 (13)	5 (16)	1.00 Ref
Lys/Gln	1 (5)	9 (19)	1.00 (0.05–18.57)	3 (8)	7 (22)	0.42 (0.06–2.48)
Gln/Gln	20 (90)	30 (62)	6.00 (0.70–51.10)	30 (79)	20 (32)	1.50 (0.38–5.85)
Lys	3 (7)	27 (28)	1.00 Ref	13 (17)	17 (27)	1.00 Ref
Gln	41 (93)	69 (72)	**5.34** (**1.52**–**18.73**)	63 (83)	47 (73)	1.75 (0.77–3.96)
*hOGG1*-Ser326Cys						
Ser/Ser	7 (32)	9 (19)	1.00 Ref	11 (29)	5 (16)	1.00 Ref
Ser/Cys	8 (36)	31 (64)	0.33 (0.09–1.16)	16 (42)	23 (72)	0.31 (0.09–1.38)
Cys/Cys	7 (32)	8 (17)	1.12 (0.27–4.63)	11 (29)	4 (12)	1.25 (0.26–5.93)
Ser	22 (50)	49 (51)	1.00 Ref	38 (50)	33 (52)	1.00 Ref
Cys	22 (50)	47 (49)	1.04 (0.51–2.12)	38 (50)	31 (48)	1.06 (0.54–2.07)
*XRCC1*-Arg194Trp						
Arg/Arg	8 (36)	12 (25)	1.00 Ref	12 (32)	8 (25)	1.00 Ref
Arg/Trp	9 (41)	22 (46)	0.61 (0.18–2.00)	14 (36)	17 (53)	0.54 (0.17–1.71)
Trp/Trp	5 (23)	14 (29)	0.53 (0.13–2.08)	12 (32)	7 (22)	1.14 (0.41–4.16)
Arg	25 (56)	46 (48)	1.00 Ref	38 (50)	33 (52)	1.00 Ref
Trp	19 (44)	50 (52)	0.70 (0.34–1.43)	38 (50)	31 (48)	1.06 (0.54–2.07)

^a^T2 vs. T3 + T4

^b^N− (node negative) vs. N+ (node positive)

## Discussion

The aim of the present study was to evaluate the associations between the risk of TNBC and polymorphisms in the genes, encoding for key proteins of BER and NER. In the present work, we analyzed three single nucleotide polymorphisms of the *XRCC1*, *hOGG1*, and *ERCC2* DNA repair genes and tested the association between the distributions of their genotypes with TNBC.

*ERCC2*-Lys751Gln, *hOGG1*-Ser326Cys, and the *XRCC1*-Arg194Trp polymorphisms have been shown to have functional significance and may be in part responsible for the interindividual difference in capacity of DNA repair in the general population and for low DNA repair efficacy in patients with various cancers [25–28].

In the presented study, *ERCC2*-Gln/Gln genotype was associated with an elevated risk of TNBC in the Polish population. There was a 5.71-fold increased risk of TNBC for *ERCC2*-Gln/Gln genotype carriers, compared with subjects with the *ERCC2*-Lys/Lys and Lys/Gln genotypes, respectively. We have also found that *ERCC2-*Lys751Gln polymorphism was related to tumor size. This result may suggest major contribution of the Lys751Gln polymorphism of the *ERCC2* gene in cancer development, but more studies performed on larger population are needed to draw a final conclusion.

It is known that the Gln/Gln homozygous variant of the *ERCC2* gene has been associated with an increased risk of lung, skin, bladder, and breast cancer [20, 21, 29, 30].

The role of *ERCC2*-Lys751Gln polymorphisms and breast cancer development is still unknown. To date, no studies have addressed the association between alterations in this region of the *ERCC2* gene and TNBC. Because a proper functioning of the *ERCC2* gene is important for the genomic stability, its alternations may be associated with higher cancer susceptibility.

Breast cancer is estrogen related. Estrogen mediates cellular growth and differentiation in tissues such as the mammary gland, endometrium, bone, cardiovascular system, brain, and urogenital tract in men and women, with the intracellular estrogen functioning as a hormone-dependent transcriptional regulator. Estrogen metabolism in eukaryotic cells includes formation of a variety of intermediate forms and production of ROS [31].

BER is very important for repairing base damage induced by ROS. In our study, we analyzed the association between polymorphisms of two genes of BER and TNBC.

In the literature, much research suggests that Ser326Cys polymorphism of *hOGG1* gene may contribute to mammary carcinogenesis [32–34]. However, the reported results have rather been inconsistent [35–37]. What is important is that recent reports introduce the role of Ser326Cys polymorphism in the development of TNBC [34].

In the recent studies, Ser326Cys polymorphism of *hOGG1* may be associated with an elevated tumor risk in the Chinese populations, regarding TNBC [28], while there are still no data, which would be illustrating the significance of *hOGG1* polymorphism for TNBC development in other populations. In the reported study, the Ser326Cys polymorphism of *hOGG1* gene was not correlated with triple-negative breast carcinoma progression.

Literature data suggest a protective role of the Trp/Trp genotype of the Arg/Trp polymorphism of the *XRCC1* gene against the development of cancer, and this function can be underlined by increasing the activity of BER [27, 38]. In the literature, many reports confirm the significance of *XRCC1*-Arg194Trp polymorphism, regarding the risk of breast carcinoma [39–42]. This is not in agreement with our result. In the present work, no significant associations were observed between Arg194Trp genotype of *XRCC1* and the incidence of TNBC in the Polish women.

In conclusion, in the present study, an association was identified between Lys751Gln polymorphism of *ERCC2* and the incidence of TNBC. The obtained data suggest that the reported study may be the first observation of the polymorphisms in *ERCC2*, *hOGG1*, and *XRCC1* genes, involved in the DNA repair pathway, to be associated with triple-negative breast carcinoma risk in the population of Polish women. Further studies, conducted on a larger group, are suggested to clarify this point.
